# Mitral valve E-Point to Septal Separation (EPSS) measurement by cardiac magnetic resonance Imaging as a quantitative surrogate of Left Ventricular Ejection Fraction (LVEF)

**DOI:** 10.1186/1532-429X-14-S1-P154

**Published:** 2012-02-01

**Authors:** Abdalla Elagha, Anthon Fuisz

**Affiliations:** 1 Cardiac MRI, Washington Hospital Center, Washington DC, DC, USA; 2 Cardiology Department, Cairo Univesrsity Hospitals, Cairo, Egypt

## Summary

The EPSS measurement by cardiac MRI is an easy and feasible method, which allows parallel assessment of LV function, in patient with and without myocardial fibrosis.

## Background

Using Echocardiography, the mitral Valve E-Point to Septal Separation (EPSS) is a straightforward approach that roughly corresponds to the status of left ventricular (LV) function, but its use has been limited to echocardiography and without solid quantitative correlation to left ventricular ejection fraction (LVEF). It may also cause underestimation of EF due to endocardial echo dropout. Cardiac MRI (CMR) has a better spatial resolution than echocardiography, and is characterized by superior endocardial border definition, facilitating more accurate assessment of structural borders. The MRI LVEF by Simpson’s method is widely considered the most accurate and most reliable method for quantifying the LVEF. Assessment of EPSS by CMR seems very attractive and simple measurement, which can be an additional standard tool in clinical MRI report for quantitative evaluation of LV function. Our objective was to test the feasibility of measuring EPSS by CMRI, and its quantitative correlation with LVEF measured by MRI Simpson’s method in heterogeneous patient groups.

## Methods

**Figure 1:**
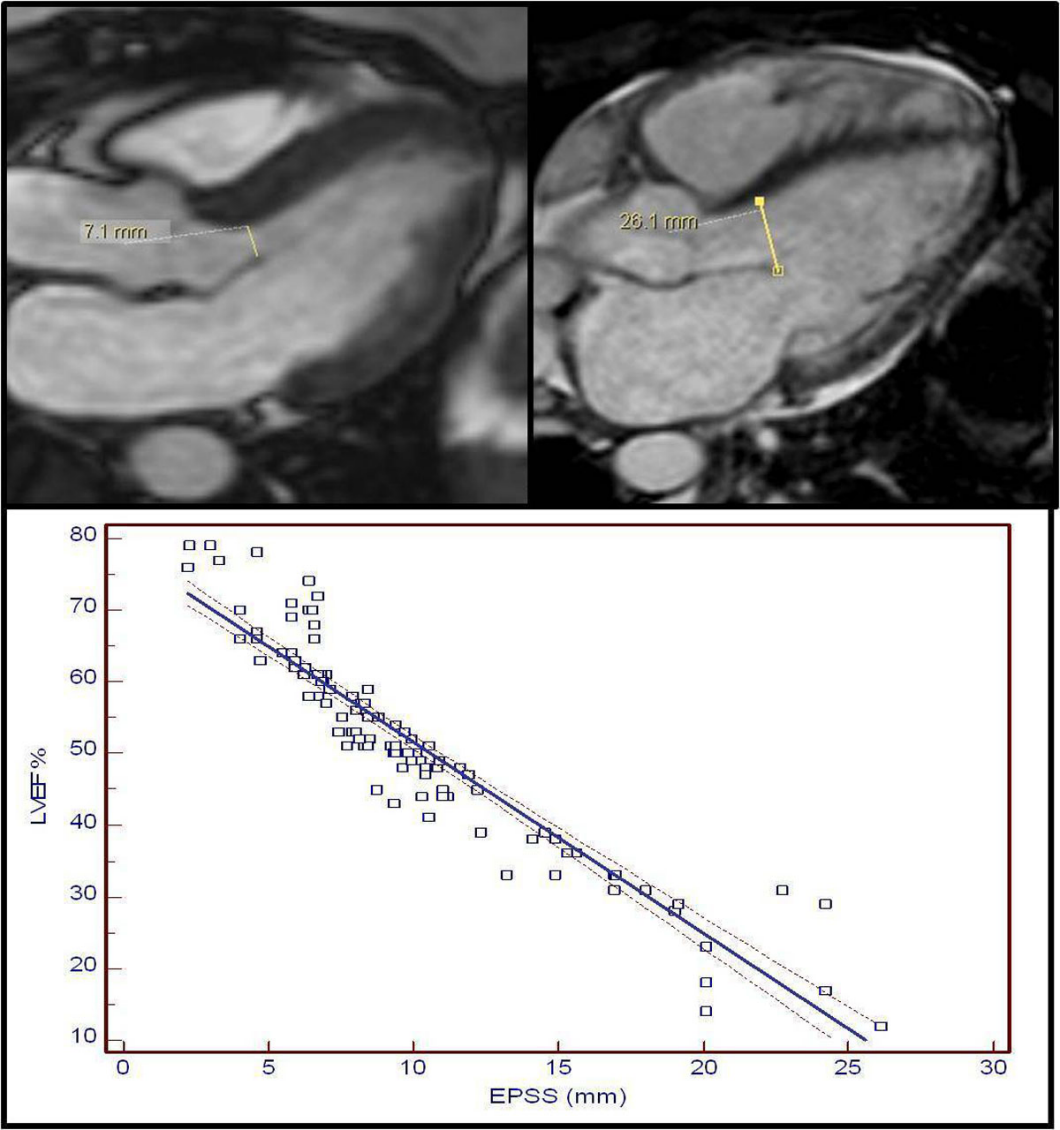
(a) Top: Measurement of EPSS in 3-chamber view (cine SSFP image). The mitral valve E-septal separation measured 7.1mm (upper left) and 26.1 mm( upper right), as shown by the line representing the minimal separation distance between the mitral valve anterior leaflet tip and the ventricular septum. The E.F. was 59 % and 12%; respectively . (b) Bottom: Scatter diagram of the MRI LVEF vs.the EPSS with regression line (solid line) and 95% confidence intervals (dotted lines).

We studied a total of 143 patients, who underwent a full CMR study. Nineteen patients with significant aortic insufficiency, mitral stenosis/prosthesis, or septal hypertrophy were excluded. Short-axis cross-sectional stack images were used to estimate LVEF by Simpson’s method. The EPSS was determined using image plane corresponding to a 3-chamber view, known as LVOT view. The EPSS was measured in millimeters (mm) as the minimal separation distance between the mitral valve anterior leaflet and the ventricular septum, usually occurring at the maximal filling phase of cardiac cycle(Figure 1a). Cautious tracking of the leaflet through diastole, frame by frame, allows measurement of shortest distance between leaflet tip and the interventricular septum. Furthermore, we divided patients into two groups according to presence or absence of fibrosis on delayed hyperenhancement (DHE) MRI study.

## Results

The LVEF ranged from 12-79 %. The EPSS ranged from 2.2-26.1 mm. We used correlation and linear regression analysis to analyze the relation between the LVEF and the EPSS. Correlation coefficient revealed to be very strong (r= -0.92; 95% Confidence interval for r= -0.95 to -0.87) with high significant level (P<0.0001). Using regression analysis, MRI LVEF could be estimated from the following equation (LVEF = 78.1569 - (2.6661 x EPSS)), with strong regression coefficient (r2= 0.86).(Figure 1b).

Also, correlation and regression coefficients were found to be closely similar in both groups with and without DHE fibrosis (r= -0.94 with no fibrosis, r= -0.91 with fibrosis; P<0.0001 for both groups).

## Conclusions

The EPSS measurement by cardiac MRI is an easy and reliable method, which allows parallel assessment of LV function, in patient with and without myocardial fibrosis. The EPSS can generate a rapid quantitative idea on LV function, especially when acquisition of multiple breath-hold short-axis images is difficult.

## Funding

Washington Hospital Center.

**Table 1:** Correlation between EF range value measured by cardiac MRI and EPSS range distance.

LVEF	EPSS range (mm)
Normal ( ≥ 60 %)	≤ 7.3
Low normal (55-59 %)	7.4-8.9
Mild systolic dysfunction (40-54%)	9-13.9
Moderate systolic dysfunction (30-39%)	14-17.1
Severe systolic dysfunction (< 30%)	>17.1

